# Investigating the current environmental situation in the Middle East and North Africa (MENA) region during the third wave of COVID-19 pandemic: urban vs. rural context

**DOI:** 10.1186/s12889-021-12313-3

**Published:** 2022-01-26

**Authors:** Mohamed Abouzid, Dina M. El-Sherif, Yahya Al Naggar, Mohammed M. Alshehri, Shaima Alothman, Hesham R. El-Seedi, Rayhana Trabelsi, Osama Mohamed Ibrahim, Esraa Hamouda Temraz, Ahmad Buimsaedah, Ibrahim Adel Aziz, Muhammad Alwan, Nuha Hadi Jasim Al Hasan, Heba Nasser Ragab, Abdullah Muhammed Koraiem, Mareb H. Ahmed, Heba Hamouda Temraz, Alyaa Khaled Madeeh, Mohanned Osama Alshareif, Fatimah Saad Elkhafeefi, Imed-Eddine Badis, Asmaa E. Abdelslam, Almajdoub Ali Mohammed Ali, Nour El Imene Kotni, Thuraya Amer

**Affiliations:** 1 Department of Physical Pharmacy and Pharmacokinetics, Poznan University of Medical Sciences, 6 Święcickiego Street, 60-781 Poznan, Poland; 2 National Institute of Oceanography and Fisheries, NIOF, Cairo, Egypt; 3 Zoology Department, Faculty of Science, Tanta University, Tanta, 31527 Egypt; 4 General Zoology, Institute for Biology, Martin Luther University Halle-Wittenberg, Hoher weg 8, 06120 Halle (Saale), Germany; 5 Physical Therapy Department, Jazan University, Jazan, Southern Region Saudi Arabia; 6 Lifestyle and Health Research Center, Health Science Research Center, Princess Nourah Bint Abdulrahman University, Riyadh, Saudi Arabia; 7 International Research Center for Food Nutrition and Safety, Jiangsu University, Zhenjiang, 212013 China; 8 Pharmacognosy Group, Department of Pharmaceutical Biosciences, BMC, Uppsala University, Box 591, 751 24 Uppsala, SE Sweden; 9 International Joint Research Laboratory of Intelligent Agriculture and Agri-products Processing, Jiangsu Education Department, Jiangsu University, Jiangsu, China; 10 EHS Maternity and Pediatrics, Touggourt, Algeria; 11 Faculty of Pharmacy, Cairo University, Cairo, Egypt; 12 College of Pharmacy, University of Sharjah, Sharjah, UAE; 13 Faculty of Medicine, Menofia University, Menofia, Egypt; 14 Aljala Hospital, Benghazi, Libya; 15 Faculty of Medicine, Al Neelian University, Khartoum, Sudan; 16 Faculty of Medicine, Aleppo University, Aleppo, Syria; 17 Department of Engineering materials, College of Engineering, University of Basrah, Basrah, Iraq; 18 Faculty of Medicine, Fayoum University, Fayoum, Egypt; 19 Faculty of Pharmacy, Al-Azhar University, Naser City, Cairo 11884 Egypt; 20 Mosul Medical College, University of Mosul, Mosul, Iraq; 21 Faculty of Medicine, Islamic University of Gaza, Gaza, Palestine; 22 SHO General Surgery Department, Aljala Hospital, Benghazi, Libya; 23 Tlemcen Faculty of Medicine, Tlemcen, Algeria; 24 Faculty of Medicine for Girls, Al-Azhar University, Cairo, Egypt; 25 Brega General Hospital (BGH), Brega, Libya; 26 School of Medicine and Health Care, Faculty of Medicine, University of Oran, Oran City, Algeria; 27 Radiography Techniques Department, Al-Turath University College, Baghdad, Iraq

**Keywords:** COVID-19, Coronavirus, Environment, Pollution, Middle East, North Africa

## Abstract

**Background:**

Coronavirus 2019 (COVID-19) pandemic led to a massive global socio-economic tragedy that has impacted the ecosystem. This paper aims to contextualize urban and rural environmental situations during the COVID-19 pandemic in the Middle East and North Africa (MENA) Region.

**Results:**

An online survey was conducted, 6770 participants were included in the final analysis, and 64% were females. The majority of the participants were urban citizens (74%). Over 50% of the urban residents significantly (*p* < 0.001) reported a reduction in noise, gathering in tourist areas, and gathering in malls and restaurants. Concerning the pollutants, most urban and rural areas have reported an increase in masks thrown in streets (69.49% vs. 73.22%, resp.; *p* = 0.003). Plastic bags and hospital waste also increased significantly with the same *p*-value of < 0.001 in urban areas compared with rural ones. The multifactorial logistic model for urban resident predictors achieved acceptable discrimination (AUROC = 0.633) according to age, crowdedness, noise and few pollutants.

**Conclusion:**

The COVID-19 pandemic had a beneficial impact on the environment and at the same time, various challenges regarding plastic and medical wastes are rising which requires environmental interventions.

**Supplementary Information:**

The online version contains supplementary material available at 10.1186/s12889-021-12313-3.

## Background

In December 2019, the first coronavirus disease 2019 (COVID-19) case was reported in China. COVID-19 was declared a global pandemic four months later, in March 2020, due to its rapid spread and severe health consequences [1]. The effect of the COVID-19 pandemic on health has prompted countries around the world to enact precautionary steps and strategies [2] such as partial or total shutdown, mandatory facemask use, social distancing, and repeated handwashing [3]. The Pandemic led to a massive global socio-economic disorder that has impacted both individuals [4], and the ecosystem directly or indirectly, such as air and water quality improvements and pollution reduction and ecological restoration [5–8]. On the contrary, the expanded usage and disposal of personal protective equipment (PPE), for instance, facial masks, hand gloves, gowns, face shields, etc., are creating environmental damage [9–11].

Lockdown and restricted travel have had mostly positive impacts on air and water quality. Several reports around the world have recorded a substantial reduction in air quality indices such as reduced concentrations of nitrogen dioxide (NO_2_) and particulate matter that have a diameter of less than 2.5 μm (PM2.5) [12, 13]. This is remarkable since air pollution causes around 3.45 million premature deaths worldwide, with international trade and transportation playing a role [14]. As a result of the production of goods (and their related pollutants) in one country for use in another, international trade contributes to the globalization of emissions and pollution [14]. Moreover, the lockdown has reduced air pollution to the point that residents of Punjab can see The Himalayas from some of their towns, despite the long distance between Punjab to The Himalayas which is more than 100 miles [15].

Further, beaches are also used as a vital economic resource for coastal areas that are threatened by pollution, mostly due to tourism. The lockdown, on the other hand, has turned the tables. With each passing day, not only is the skyline getting brighter, but the waterways are becoming visibly purer, and the once-endangered flora and fauna are now coming back to life indicating how the Earth has been healing since the lockdown [15]. Thus, as a result of the COVID-19 restriction on travel and beaches around the world have been reporting improvement on their environmental indices [12]. Ganga can be cleaner today than in 1986, the year the first attempts at cleaning the river were initiated, according to a report published by Hindustan Times. The Yamuna has a similar scenario; a cleaner Yamuna is noticeable because of a blanket reduction in agricultural pollution and improved water discharge from Haryana to Delhi. The auto purification of the river has been improved by both influences. Pink flamingoes returned in huge numbers to Mumbai beach. The reduction in the intensity of human activities at and around the city is being touted as a major reason for the possibility of flamingos flocking to the city in such large numbers [15].

However, due to the ongoing COVID-19 epidemic, the usage and disposal of face masks, gloves, face shields, and other forms of PPE has grown considerably. Many countries require the use of PPE as an effective and low-cost method of reducing viral transmission. This, however, may represent a new challenge to solid waste management and increase plastic pollution [16]. According to a recent report, 1.56 billion face masks are likely to enter the oceans in 2020 [17]. Recent research has found various types of PPE in South American coastal cities [18], African lakes and beaches [19], and European cities [20].

Examining and understanding the environmental effects of COVID-19 precautionary measures in combination with people’s behavior toward their environment is crucial for stakeholders to develop and implement necessary policies to protect the environment. However, the practices and perceptions of people living in the Middle East and Northern Africa (MENA) region about the effect of COVID-19 on their environmental parameters (air and water quality, and medical waste production and recycling) are poorly understood. Thus, the primary objective of this study is to investigate the current environmental situation during the COVID-19 pandemic in the MENA region. A secondary objective is to contextualize urban vs. rural environmental situations.

## Methods

An online survey was conducted in nine countries (Egypt, Algeria, Libya, Sudan, Saudi Arabia, Emirates, Syria, Palestine, and Iraq) from the MENA region in April and May 2021. The questionnaire included observational information on environmental status and multiple pollutants during the COVID-19 period. Multiple logistic regression was performed to determine urban residents’ predictors.

### Participants

**Fig. 1:**
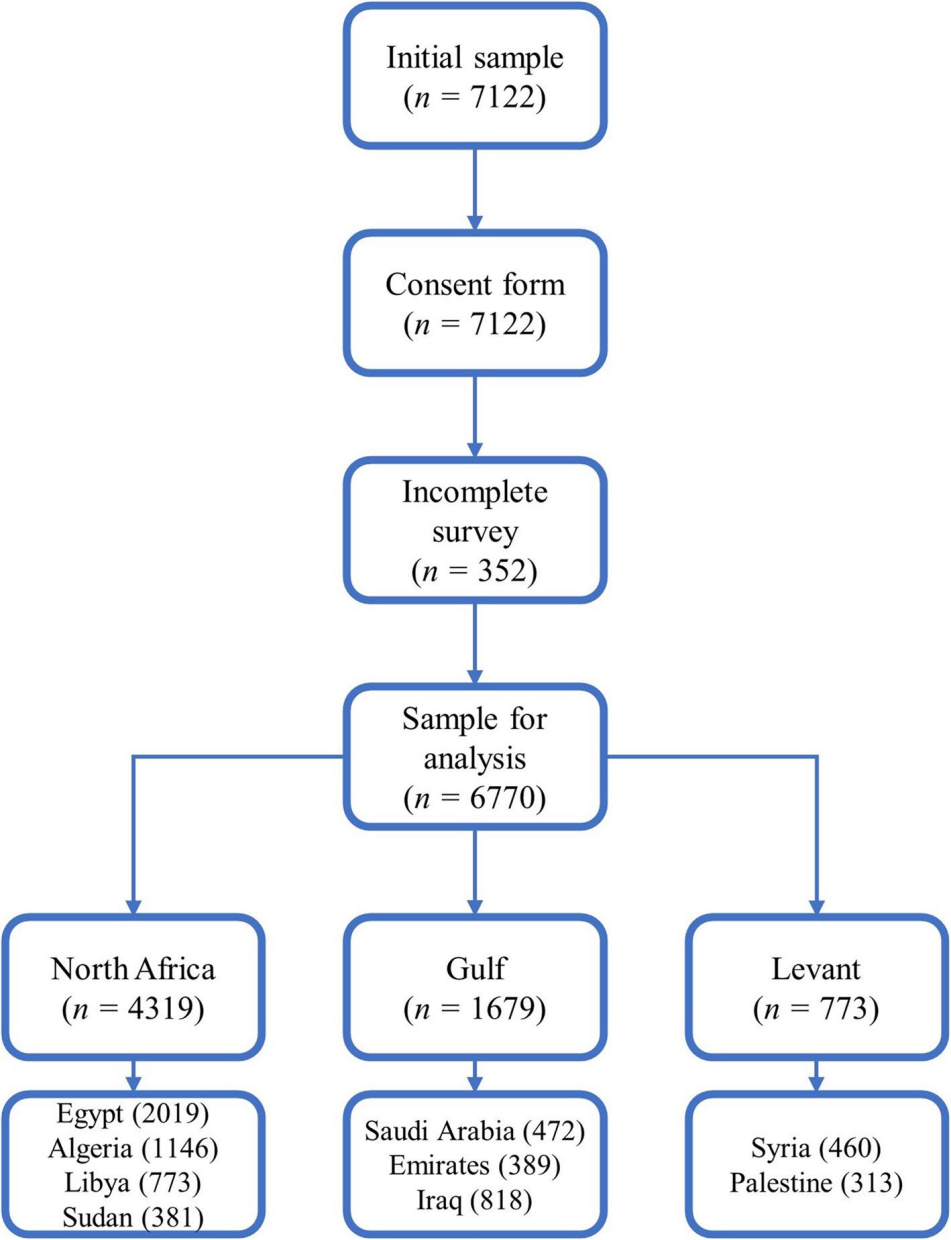
Inclusion/exclusion process of the responses

Participants had to be at least 18 years old, residing in any MENA region country through COVID-19 pandemic, speaking Arabic or English, and had to fill the entire survey confirming the consent to participate in the study. The current study’s participants were residing in 9 countries, and incomplete responses were excluded (see Fig. 1 for inclusion/exclusion process). Participants’ demographic characteristics appear in Table 1.

**Table 1:** Demographic characteristics of the respondents

Demographic characteristics^a^	
Age	
18-25	3339 (49.3)
26-35	1754 (25.9)
36-45	1005 (14.8)
46-60	531 (7.8)
> 60	142 (2.1)
Gender	
Female	4320 (63.8)
Male	2451 (36.2)
Country	
Egypt	2019 (29.8)
Algeria	1146 (16.9)
Libya	773 (11.4)
Sudan	381 (5.6)
Saudi Arabia	472 (7)
Emirates	389 (5.7)
Syria	460 (6.8)
Palestine	313 (4.6)
Iraq	818 (12.1)
Living area	
Rural	1760 (26)
Urban	5011 (74)
Level of education	
Uneducated	331 (4.9)
High school/technical school	974 (14.4)
Bachelor’s or equivalent	4376 (64.6)
Master or doctoral	1090 (16.1)

^a^ Data reported as n (%)

### Questionnaire formulation and validation

To develop the questionnaire, a broad inspection of related review articles was performed. The first edition of the survey (Supplementary file 1) was evaluated by a panel of experts (*n* = 6; mentioned in the acknowledgment section). The final version of the questionnaire (Supplementary file 1) was composed of three domains: (I. six demographic questions; II. seven “1-5 Likert scale” questions for measuring the impact of COVID-19 on the environment; III. eight “1-5 Likert scale” questions for measuring the impact of COVID-19 on pollution increase) (α = 0.81 and 0.73 for domain II and III respectively; Supplementary file 2).

### Procedure

This study is the primary analysis of cross-sectional survey data. The survey was available in two languages (English and Arabic), and it was hosted on Google Forms. The collaborators were responsible for distributing the survey using social media and mailing lists. Participation was voluntary - participants did not receive any monetary compensation for their participation.

### Statistical analysis

Data were analyzed using IBM SPSS Statistics (version 26) and TIBCO Statistica (version 13; equipped with Medical Bundle version 4.0.67). Categorical data were reported as frequency/percentage and continuous data as mean/standard deviation. Normality was calculated using Shapiro-Wilk tests. Differences between rural and urban results were calculated by unpaired sample *t*-test and confirmed by Mann–Whitney *U* test and Fisher test, this statistical approach to analyze Likert scale data was applied in previous studies [21, 22]. Moreover, a multi logistic regression model was performed to study the urban residents’ predictors. Logistic regression results were presented as odds ratios (ORs) and 95% confidence interval (95% CI). *p*-value < 0.05 is considered statistically significant for all the results.

## Results

A total of 6770 participants were included in the final analysis, 64% were females and 36% were males. The majority of them were urban citizens 74%. Participation between the ages of 18 and 25 had the highest percentage of participants (49%) compared to only 2% of those over 60.

### Estimating the association between COVID-19 and environmental factors

Over 50% of the urban residents reported a significant reduction in the level of noise, gathering in tourist areas, and crowding in malls and restaurants. Moreover, 47% reported an improvement in the air quality, and a reduction in gas emission from factories compared with almost 40% in rural areas. Crowdedness also was significantly reduced in urban areas as reported by 46% of the residents. Despite only 38% in urban areas reported an improvement in water quality in rivers and lakes, the result remains significant in comparison with rural areas with a value of 32%. All the items in this domain were significant at (*p* < 0.001). Comparison between the environmental status in rural and urban areas is represented in (Table 2).

**Table 2:** Respondents’ perceptions of the environmental status in rural and urban areas

	Rural *n* = 1760			Urban *n* = 5011			Unpaired *t*-test	95% Confidence Interval		Mann-Whitney *U* test^γ^		Fisher’s exact test^δ^
	Mean (SD)*	No. (%) disagree^ω^	No. (%) agree^α^	Mean (SD)*	No. (%) disagree﻿^ω^	No. (%) agree^α^	*p*-value^β^	lower	Upper	*Z*-value	*p*-value	
Did you notice an improvement in the air quality?	3.21 (0.99)	411 (23.36%)	682 (38.75%)	3.38 (0.99)	912 (18.2%)	2353 (46.96%)	< 0.001	0.111	0.219	−6.288	< 0.001	< 0.001
Did you notice a decrease in factory gas emissions?	3.25 (0.99)	355 (20.18%)	697 (39.6%)	3.38 (1.03)	1003 (20.02%)	2374 (47.38%)	< 0.001	0.073	0.182	−4.705	< 0.001	< 0.001
Did you notice an improvement in the water quality of rivers and lakes?	3.01 (1.06)	579 (32.9%)	564 (32.05%)	3.17 (1.03)	1275 (25.45%)	1880 (37.52%)	< 0.001	0.102	0.216	−5.656	< 0.001	< 0.001
Did you notice a decrease in the surrounding noise?	3.22 (1.24)	636 (36.14%)	886 (50.34%)	3.55 (1.22)	1291 (25.77%)	3143 (62.72%)	< 0.001	0.265	0.400	−9.866	< 0.001	< 0.001
Did you notice the numbers decreased in the tourist areas?	3.7 (1.01)	219 (12.45%)	1119 (63.58%)	3.84 (1.04)	664 (13.26%)	3628 (72.4%)	< 0.001	0.085	0.195	−6.184	< 0.001	< 0.001
Did you notice that the numbers and gatherings in malls, restaurants, and stores decrease?	3.02 (1.37)	791 (44.95%)	821 (46.65%)	3.35 (1.37)	1788 (35.69%)	2941 (58.69%)	< 0.001	0.254	0.402	−8.725	< 0.001	< 0.001
Did you notice that public transport is less crowded?	2.53 (1.38)	1075 (61.08%)	567 (32.22%)	2.97 (1.42)	2321 (46.32%)	2309 (46.08%)	< 0.001	0.361	0.513	−11.070	< 0.001	< 0.001

* – Participant perceptions were measured using the following scale: 1 = Strongly Disagree; 2 = Disagree; 3 = Neither Agree nor Disagree; 4 = Agree; 5 = Strongly Agree

ω– Percentage in disagreement was calculated using those who responded “Strongly disagree” or “Disagree”

^α^ – Percentage in agreement was calculated using those who responded “Strongly agree” or “Agree”

^β^ – *p*-values from unpaired *t*-test comparing rural with urban (assuming unequal variance)

^γ^ – Used to confirm unpaired *t*-test results

^δ^ – Used to compare No. (%) agree between rural with urban

### Estimating the association between COVID-19 and pollution level

Based on the obtained responses, there is a significant increase (*p* = 0.003) in the number of masks thrown in the street in both urban and rural areas. Although only 33 and 29% of the participants reported an increase in the aforementioned waste in rivers and lakes in urban and rural areas, respectively, the increase is still significantly different (*p* = 0.001). No significant increase in the number of plastic gloves has been noticed in rivers or lakes, while plastic bags were shown to be more likely thrown in streets in the urban area compared with rural (*p* < 0.001). Half of the respondents reported a significant increase (*p* < 0.001) in hospital waste in urban areas compared with 45% in rural ones. While the variations in laboratory waste increase and places designated for medical wastes between rural and urban areas were not significant (*p* = 0.077). Pollution indicators for urban and rural areas are shown in (Table 3).

**Table 3:** Respondents’ perceptions of the pollution indicators between rural with urban

	Rural, *n* = 1760			Urban *n* = 5011			Unpaired *t*-test	95% Confidence Interval		Mann-Whitney *U* test^γ^		Fisher’s exact test^δ^
	Mean (SD)*	No. (%) disagree﻿^ω^	No. (%) agree^α^	Mean (SD)*	No. (%) disagree﻿^ω^	No. (%) agree^α^	*p*-value^β^	lower	Upper	*Z*-value	*p*-value	
Did you notice an increase in the number of masks thrown on the streets?	3.64 (1.13)	362 (20.57%)	1223 (69.49%)	3.78 (1.09)	850 (16.97%)	3669 (73.22%)	< 0.001	0.077	0.199	−4.605	< 0.001	0.003
Did you notice an increase in the number of masks thrown in rivers and lakes?	3.13 (0.93)	376 (21.37%)	517 (29.38%)	3.22 (0.96)	952 (19%)	1665 (33.23%)	0.001	0.036	0.138	−3.348	< 0.001	0.003
Did you notice an increase in the number of plastic gloves thrown on the streets?	2.89 (1.19)	817 (46.43%)	663 (37.67%)	3.12 (1.23)	1939 (38.7%)	2341 (46.72%)	< 0.001	0.166	0.296	−6.849	< 0.001	< 0.001
Did you notice an increase in the number of plastic gloves thrown in rivers and lakes?	2.96 (0.98)	516 (29.32%)	413 (23.47%)	3.02 (1)	1346 (26.87%)	1327 (26.48%)	0.016	0.012	0.119	−2.578	0.010	0.013
Did you notice an increase in the number of plastic bags thrown on the street?	3.29 (1.08)	456 (25.91%)	850 (48.3%)	3.44 (1.09)	1184 (23.63%)	2718 (54.24%)	< 0.001	0.092	0.209	−4.976	< 0.001	< 0.001
Did you notice an increase in hospital waste?	3.45 (0.96)	229 (13.02%)	797 (45.28%)	3.55 (1)	643 (12.84%)	2543 (50.75%)	< 0.001	0.053	0.158	−4.013	< 0.001	< 0.001
Did you notice an increase in laboratories’ wastes?	3.4 (0.94)	224 (12.73%)	745 (42.33%)	3.45 (0.95)	617 (12.32%)	2209 (44.08%)	0.077	−0.005	0.098	−1.614	0.106	0.209
Did you notice an increase in special places designated for medical waste disposal?	3.02 (1.06)	508 (28.87%)	534 (30.34%)	3.04 (1.1)	1453 (29%)	1640 (32.73%)	0.372	−0.032	0.085	−0.999	0.318	0.066

* – Participant perceptions were measured using the following scale: 1 = Strongly Disagree; 2 = Disagree; 3 = Neither Agree nor Disagree; 4 = Agree; 5 = Strongly Agree

ω – Percentage in disagreement was calculated using those who responded “Strongly disagree” or “Disagree”

^α^ – Percentage in agreement was calculated using those who responded “Strongly agree” or “Agree”

^β^ – p-values from unpaired *t*-test comparing rural with urban (assuming unequal variance)

^γ^ – Used to confirm unpaired *t*-test results

^δ^ – Used to compare No. (%) agree between rural with urban

### A multifactorial model for urban resident predictors

**Fig. 2:**
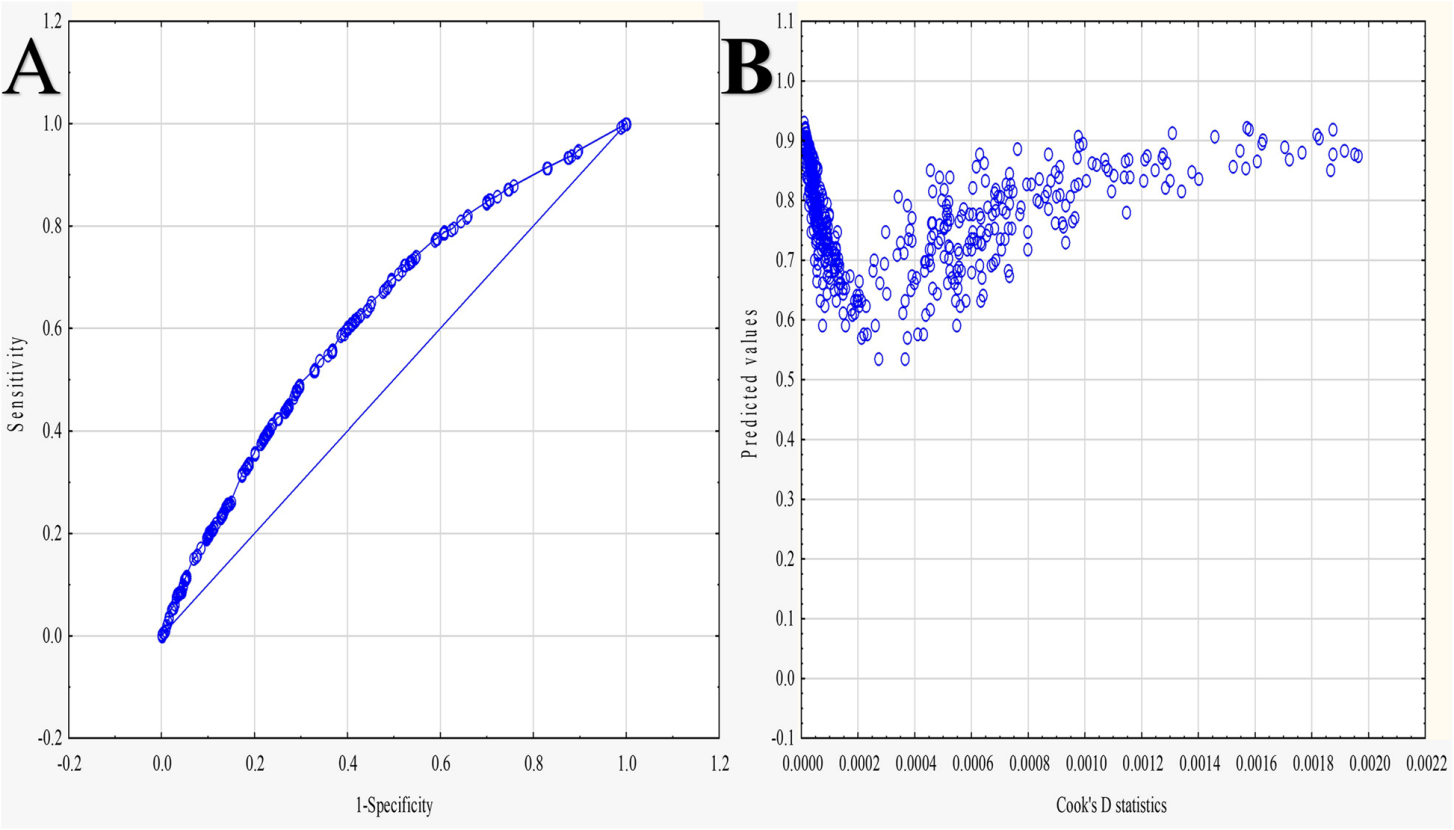
**A** The area under the receiver operating characteristics (AUROC = 0.633); **B** cook’s D statics and influential outliers  ≥ 0.0006

According to the information collected, 74% of the respondents were resident in urban areas, and this was associated with higher age (OR = 1.37; 95% CI = 1.29–1.46; *p* < 0.001), increase in the number of plastic gloves (OR = 1.15; 95% CI = 1.02–1.29; *p* = 0.026) and plastic bags thrown in the street (OR = 1.18; 95% CI = 1.04–1.35; *p* = 0.014), respectively. However, among those participants, a reduction in noise (OR = 1.25; 95% CI = 1.11–1.42; *p* < 0.001), gathering in touristic areas (OR = 1.18; 95% CI = 1.04–1.34; *p* = 0.01), and crowdedness in the public transportation (OR = 1.31; 95% CI = 1.14–1.50; *p* < 0.001) were noticed. Observing the increase in the number of plastic gloves in rivers and lakes was inversely associated with urban residents (OR = 0.80; 95% CI = 0.68–0.93; *p* = 0.004). The Multifactorial model of the significant urban resident predictors is visible in Table 4. The area under the receiver operating characteristics (AUROC) value was 0.633, and 0.7 ≥ AUROC > 0.6 indicates acceptable discrimination [23] (Fig. 2). Moreover, results of cook’s D statics and influential outliers are visible in Fig. 2 [24].

**Table 4:** Multifactorial model by backward stepwise regression shows urban resident predictors

	Estimate	Standard error	Wald test	95% Confidence interval		*p*	Odds ratio	95% Confidence odds ratio	
				Lower	Upper			Lower	Upper
Age	0.316	0.031	101.932	0.255	0.377	0.000	1.372	1.290	1.458
Did you notice an increase in the number of plastic gloves thrown on the street?	0.135	0.061	4.985	0.017	0.254	0.026	1.145	1.017	1.289
Did you notice an increase in the number of plastic gloves thrown in rivers and lakes?	−0.227	0.078	8.370	−0.381	−0.073	0.004	0.797	0.684	0.929
Did you notice a decrease in the surrounding noise?	0.226	0.064	12.631	0.101	0.350	0.000	1.253	1.107	1.420
Did you notice the numbers decreased in the tourist areas?	0.167	0.064	6.712	0.041	0.293	0.010	1.181	1.041	1.340
Did you notice an increase in the number of plastic bags thrown on the streets?	0.169	0.068	6.097	0.035	0.303	0.014	1.184	1.035	1.354
Did you notice that public transport is less crowded?	0.266	0.069	14.866	0.131	0.401	0.000	1.305	1.140	1.494

## Discussion

The COVID-19 pandemic is posing a severe threat to nations around the world since entire populations have succumbed to the disease’s spread and have resorted to social isolation. Here we aimed to see how the third wave of the COVID-19 pandemic (April-May 2021) affected the environment in the MENA region, as well as to compare urban and rural environmental settings.

People in the MENA region reported a reduction in noise and pollution, and an improvement in the air quality, especially in urban areas, as an industry, transportation, and companies partially or totally shut down during the pandemic. Google’s Community Mobility Reports for Egypt, Iraq, Libya, Saudi Arabia, and Yemen during the observed period are available in (Supplementary file 3). Similar findings have been reported in various parts of the world. For example, due to the suspension of heavy factories in China, NO_2_ and carbon oxide (CO) levels have decreased by almost half [25]. Moreover, according to Le et al., the lockdown in the urban areas located in northern China has decreased the various emissions up to 90% resulted in ozone (O_3_) enhancement in these regions [26]. In India, there was a reduction in surface temperature, tropospheric NO_2_ density and O_3_, which displayed a significant improvement in Air Quality Index [27]. The European Environmental Agency (EEA) reported that the COVID-19 shutdown lowered NO_2_ emissions by 30-60% in several European cities, including Barcelona, Madrid, Milan, Rome, and Paris [28]. Rodríguez-Urrego and Rodríguez-Urrego have recently summarized the overall status of PM2.5 in the 50 most contaminated capitals and found an average reduction of by 12% in PM2.5. According to them, Bogotá city in Colombia showed the highest PM2.5 reduction with values of 57% [29]. As a result, a direct or indirect impact on the environment has been documented, such as better air and water quality, noise reduction, and ecological restoration [5–7]. It is worth mentioning that the time of measurement was essential, as the short-term lockdown did not affect air quality in New York City at the beginning of the pandemic [30]. However, recent results show considerable PM2.5 reduction followed by Substantial health and associated economic benefits [31]. Unlike the majority of urban areas, the quality of the air in rural areas did not change such as in Gadanki, India [32]. Martorell-Marugán et al. have also confirmed that the lockdown impact on rural air quality is smaller than in urban environments [33], which also has been noticed in our study since only 38.75% of the rural area citizens have reported an improvement in the quality of the air.

Noise reduction was reported in urban areas by other studies such as in Dublin [34], Boston [35], Rio de Janeiro [36], and Madrid [37]. The reduction of noise pollution had many reasons such as restricted access to parks and main stations and the absence of people and techniques in main streets. Similar results were reported in Maharashtra State in India, despite the festival culture in that State. However, the reduction in the noise was due to the implementation of an eco-friendly way of celebrating by the authorities. Contradictory results were published by Tong et al. [38], during the lockdown in London, there was a significant increase in noise due to complaints. Moreover, they found that noise complaints were higher in areas with higher unemployment rates, more residents with no qualifications and lower house prices.

Similar to what we found in our study, the level of crowding in tourist and commercial areas reduced in the tourist spots around the world due to the outbreak of COVID-19 and local restrictions [12]. The local authority, for example, imposed a restriction on public gatherings and visitor arrivals at Cox’s Bazar Sea beach, the world’s longest uninterrupted natural sand sea beach. As a result of the restriction, the color of the seawater changes, which is typically muddy due to swimming, bathing, playing, and riding motorized boats [39]. Due to the absence of industrial pollutants during India’s lockdown days, the rivers Ganga and Yamuna have attained a remarkable level of purity [8]. Other studies have reported an increase in other types of crowding that was associated with negative outcomes on public health such as nursing home crowding [40], informal urban settlements [41], and household crowding [42].

One of the most serious issues that arose as a result of the COVID-19 lockdown, as reported in the current study and other studies, is the increased use of facial masks, hand gloves, gowns, and face shields, as well as the creation of a large volume of hospital waste containing plastic materials. Ryan et al. have reported an increase in plastic waste due to single-use hygiene products such as cotton wool and wet wipes in Durban streets [43]. Similar studies stated the increase of PPE in streets, rivers and beaches [2, 20, 44]. Moreover, a statistical model has been designed by Abu-Qdais et al. confirming a significant increase in medical waste in the King Abdullah University Hospital in Jordan; the hospital had 95 patients with COVID-19 and was producing daily almost 650 kg as medical waste [45]. Other medical facilities in China and Spain have reported an extreme increase in medical waste with values of 370 and 350%, respectively [46]. Medical waste is considered a threat to public health, therefore, some countries require medical waste to be incinerated using high temperatures which can lead to the release of greenhouse gas, as well as other potentially dangerous compounds, such as heavy metals, dioxins, polychlorinated biphenyls and furans [47]. Therefore, plastic and microplastic pollution are trending topics since the majority of medical wastes are made of plastic [48, 49]. China has provided an alternative solution for the treatment of the medical waste, for example, 200 tons of medical waste was produced by Wuhan inhabitants in China in a single day which is four times higher than the incineration ability of the city, hence the authorities deploy mobile treatment facilities [7]. Contrarywise this, few Indian cities are depending on traditional strategies such as landfilling filling and local burning [46, 50]. Therefore, as a necessary step, governments must develop and implement solutions such as the redesign of eco-friendly PPE [44] or various recycling techniques of plastics [51]. Meanwhile, while scientists are developing vaccines [52], applying new methods for SARS-CoV-2 surveillance [53], and its elimination from water systems [54]; it is everyone’s responsibility to follow the rules when disposing of their face masks and other medical waste [55]. 

Finally, it is critical to point out the limitations of our research. First, the elements affecting pollution and environmental status were not explicitly measured; instead, they were self-reported, which could lead to bias and misreporting. Second, in some countries, the data collection period was connected with partial lockdown; as a result, the participant’s observation may not be accurate owing to limited outgoing and may have been influenced by other external variables such as media, family, and friends’ perspectives. Also, even though the number of participants may be representative of each country, it may not be representative of urban or rural areas in some countries. Finally, data representing the MENA region and individual country analysis may provide different results. The strengths of this study, however, are the large amount of data collected and the high quality of the data since we avoided mass distribution; country coordinators advised respondents to distribute the questionnaire to colleagues and trusted individuals. As a result, the data can be used as a source of knowledge for the examined region’s environmental policy.

## Conclusion

In urban areas, the COVID-19 pandemic was associated with a higher positive impact on the environment compared with rural areas, such as noise and crowdedness reduction. However, pollution risk was more prevalent in urban areas. The main sources of pollution were plastic masks and bags, and hospital wastes. Environmental interventions are required to address the pollution issues raised in urban areas during the COVID-19 pandemic. Further studies should be conducted to confirm the current results using administrative data.

## Supplementary Information

**Additional file 1.**

**Additional file 2.**

**Additional file 3.**

## Data Availability

All data generated or analyzed during this study are included in this published article (Supplementary file 1: Questionnaire; Supplementary file 2: Survey Development; Supplementary file 3: Google Mobility Reports). Original dataset/raw data are available from the corresponding author on reasonable request.
