# Supplementary material for: Investigating the current environmental situation in the Middle East and North Africa (MENA) region during the third wave of COVID-19 pandemic: urban vs. rural context
Source: BMC Public Health. 2022 Jan 26;22:177. doi: 10.1186/s12889-021-12313-3 (PMC8790551; doi:10.1186/s12889-021-12313-3)
Supplement: Supplementary file 1 — Additional file 1. [file 12889_2021_12313_MOESM1_ESM.pdf]

## FINAL QUESTIONNAIRE

### Domain I: Demographic data

| No. | Question |
|---|---|
| **Q1** | Age |
| **Q2** | Gender<br>• Male<br>• Female |
| **Q3** | Level of education<br>• Uneducated<br>• High school/technical school<br>• Bachelor's or equivalent<br>• Master or doctoral |
| **Q4** | Living area<br>• Rural<br>• Urban |
| **Q5** | Country<br>• Egypt<br>• Algeria<br>• Libya<br>• Sudan<br>• Saudi Arabia<br>• Emirates<br>• Syria<br>• Palestine<br>• Iraq<br>• Oman<br>• Morocco<br>• Kuwait<br>• Lebanon<br>• Qatar<br>• Jordan<br>• Yemen<br>• Tunisia |
| **Q6** | Have you or any of your household have been infected with COVID-19?<br>• Yes<br>• No |

### Domain II: The impact of COVID-19 on the environment

***Likert scale (Strongly Disagree; Disagree; Neither Agree nor Disagree; Agree; Strongly Agree)***

| | |
|---|---|
| **Q7** | Did you notice an improvement in the air quality? |
| **Q8** | Did you notice a decrease in factory gas emissions? |
| **Q9** | Did you notice an improvement in the water quality of rivers and lakes? |
| **Q10** | Did you notice a decrease in the surrounding noise? |
| **Q11** | Did you notice the numbers decreased in the tourist areas? |
| **Q12** | Did you notice that the numbers and gatherings in malls, restaurants, and stores decrease? |
| **Q13** | Did you notice that public transport is less crowded? |

**Domain III: The impact of COVID-19 on pollution increase**

***Likert scale (Strongly Disagree; Disagree; Neither Agree nor Disagree; Agree; Strongly Agree)***

| | |
|---|---|
| **Q14** | Did you notice an increase in the number of masks thrown on the streets? |
| **Q15** | Did you notice an increase in the number of masks thrown in rivers and lakes? |
| **Q16** | Did you notice an increase in the number of plastic gloves thrown on the streets? |
| **Q17** | Did you notice an increase in the number of plastic gloves thrown in rivers and lakes? |
| **Q18** | Did you notice an increase in the number of plastic bags thrown on the street? |
| **Q19** | Did you notice an increase in hospital waste? |
| **Q20** | Did you notice an increase in laboratories' wastes? |
| **Q21** | Did you notice an increase in special places designated for medical waste disposal (masks - gloves - hospital waste -laboratory waste)? |
