# Supplementary material for: Investigating the current environmental situation in the Middle East and North Africa (MENA) region during the third wave of COVID-19 pandemic: urban vs. rural context
Source: BMC Public Health. 2022 Jan 26;22:177. doi: 10.1186/s12889-021-12313-3 (PMC8790551; doi:10.1186/s12889-021-12313-3)
Supplement: Supplementary file 2 — Additional file 2. [file 12889_2021_12313_MOESM2_ESM.pdf]

1. **Survey validation**

items	Numbers of questions in each item	Cronbach's Alpha	Cronbach's Alpha Based on Standardized Items
Domain II	7	0.811	0.813
Domain III	8	0.728	0.731
Total items	15	0.819	0.818

1.1.Domain II: the impact of COVID-19 on the environment

Questions	Mean	SD	alpha
**Q7**	3.333	0.992	0.787
**Q8**	3.346	1.024	0.789
**Q9**	3.125	1.042	0.785
**Q10**	3.466	1.235	0.773
**Q11**	3.803	1.03	0.806
**Q12**	3.264	1.376	0.778
**Q13**	2.856	1.421	0.778
**Reliability alpha**	0.811		
**Standardized alpha**	0.813		

1.2.Domain III: the impact of COVID-19 on pollution increase

Questions	Mean	SD	alpha
**Q14**	3.744	1.102	0.728
**Q15**	3.193	0.953	0.692
**Q16**	3.058	1.222	0.68
**Q17**	3.006	0.993	0.678
**Q18**	3.405	1.089	0.702
**Q19**	3.524	0.988	0.7
**Q20**	3.438	0.951	0.7
**Q21**	3.035	1.09	0.722
**Reliability alpha**	0.728		
**Standardized alpha**	0.731		

1.3. Total survey

Questions	Mean	SD	alpha
**Q7**	3.333	0.992	0.806
**Q8**	3.346	1.024	0.805
**Q9**	3.125	1.042	0.805
**Q10**	3.466	1.235	0.802
**Q11**	3.803	1.03	0.813
**Q12**	3.264	1.376	0.803
**Q13**	2.856	1.421	0.798
**Q14**	3.744	1.102	0.815
**Q15**	3.193	0.953	0.809
**Q16**	3.058	1.222	0.804
**Q17**	3.006	0.993	0.807
**Q18**	3.405	1.089	0.817
**Q19**	3.524	0.988	0.817
**Q20**	3.438	0.951	0.817
**Q21**	3.035	1.09	0.814
**Reliability alpha**	0.819		
**Standardized alpha**	0.818		

2. **Sample Size calculation**

To calculate the sample size for the pilot study, the following equation was used.

$$\boldsymbol{n_0 = \frac{z^2 \times p(1-p)}{e^2}}$$

$\boldsymbol{n_0}$	-	Sample size, which was estimated
$\boldsymbol{z^2}$	-	The selected critical value of the desired level of confidence or risk
$\boldsymbol{p}$	-	The estimated proportion of an attribute that is present in the population or maximum variability of the population
$\boldsymbol{e}$	-	The desired level of precision or margin of error

In our case, since the population number is unknown.

$\boldsymbol{n_0}$	-	?
$\boldsymbol{z^2}$	-	95% confidence level (The value of (1-α) in Standard Normal Distribution **z**-table, which is 1.96 for 95%)
$\boldsymbol{p}$	-	50% variability of the population (which is maximum)
$\boldsymbol{e}$	-	5% margin of error

Put the value in the given formula-

$$\boldsymbol{n_0 = \frac{(1.96)^2 \times 0.5(1-0.5)}{(0.05)^2} = 384}$$
